# YTHDF1’s grip on CRC vasculature: insights into LINC01106 and miR-449b-5p-VEGFA axis

**DOI:** 10.1186/s12935-024-03360-y

**Published:** 2024-06-04

**Authors:** Rui-ting Ma, Yuanyuan Wang, Feng Ji, Jian-nan Chen, Tian-jun Wang, Yan Liu, Ming-xing Hou, Zhi-gang Guo

**Affiliations:** 1 Jiangsu Key Laboratory for Molecular and Medical Biotechnology, College of Life Sciences, Nanjing Normal University, Nanjing, Jiangsu 210023 China; 2 The Affiliated Hospital of Inner Mongolia Medical University, No.1, North Channel Road, Huimin District, Hohhot, 010050 China; 3 Nanjing Medical University, Nanjing, Jiangsu 210097 China; 4 The Academy of Life Sciences, Nanjing Normal University, Nanjing, 210097 China

**Keywords:** Colorectal cancer, Linc01106, Ythdf1, m6a modification, Mir-449b-5p, Vascular generation

## Abstract

**Background:**

Investigating the unexplored territory of lncRNA m6A modification in colorectal cancer (CRC) vasculature, this study focuses on LINC01106 and YTHDF1.

**Methods:**

Clinical assessments reveal upregulated LINC01106 promoting vascular generation via the miR-449b-5p-VEGFA pathway.

**Results:**

YTHDF1, elevated in CRC tissues, emerges as an adverse prognostic factor. Functional experiments showcase YTHDF1’s inhibitory effects on CRC cell dynamics. Mechanistically, Me-CLIP identifies m6A-modified LINC01106, validated as a YTHDF1 target through Me-RIP.

**Conclusions:**

This study sheds light on the YTHDF1-mediated m6A modification of LINC01106, presenting it as a key player in suppressing CRC vascular generation.

**Supplementary Information:**

The online version contains supplementary material available at 10.1186/s12935-024-03360-y.

## Background

Colorectal cancer (CRC) is one of the most common malignant tumors worldwide, with high incidence and mortality rates compared to other cancers [1–3]. According to global cancer statistics, the number of diagnosed CRC cases continues to rise, posing a significant public health concern [4–6]. The occurrence of CRC is influenced by various factors, including genetics, environment, and lifestyle [7–9]. Although treatment methods have improved in recent years, long-term survival rates for CRC patients remain unsatisfactory [10–12]. Standard treatment options, including surgery, radiotherapy, chemotherapy, and targeted therapy, often come with risks of side effects and recurrence [13, 14]. Therefore, studying the molecular mechanisms of CRC, identifying new targets, and developing novel treatment strategies are crucial for improving treatment outcomes and survival rates in CRC patients [15–17].

Long non-coding RNAs (lncRNAs) are RNA molecules exceeding 200 nucleotides in length, lacking protein-coding capacity but playing a vital role in gene expression regulation [18–20]. Recent research has discovered the significant roles of lncRNAs, particularly in cancer onset and progression [21, 22]. lncRNAs participate in cancer development through various mechanisms, such as influencing chromosome structure, regulating gene transcription and translation, and participating in cell signaling [23, 24]. Additionally, lncRNAs are associated with tumor invasion, metastasis, and drug resistance [25, 26]. m6A modification is a common internal RNA modification that regulates RNA activity by altering its structure and function [27]. Abnormal m6A modification in cancer can result in gene expression dysregulation, promoting tumor development [28–30]. Therefore, a comprehensive understanding of lncRNA’s m6A modification in CRC is of significant importance in unraveling the molecular mechanisms of tumors and developing new therapeutic strategies [31].

YTHDF1 is a protein that recognizes m6A modification sites and regulates RNA metabolism and function by binding to m6A-modified RNA molecules [32–34]. In cancer, the expression level of YTHDF1 is closely correlated with tumor occurrence, progression, and prognosis [35, 36]. For instance, YTHDF1 overexpression is associated with aggressive tumor behavior and poor prognosis in certain cancer types [37, 38]. m6A modification plays an important role in tumor microenvironment regulation, particularly in tumor angiogenesis [39–41]. Angiogenesis is a critical process for tumor growth and metastasis, and m6A modification regulates this process by affecting the expression of related genes [42, 43]. Therefore, investigating the role of YTHDF1 in CRC, particularly how it influences angiogenesis and the tumor microenvironment through m6A modification, is of great significance for a deeper understanding of CRC’s molecular mechanisms and the identification of new therapeutic targets [44, 45].

LINC01106 is a long non-coding RNA that has been found to play a significant role in the occurrence and progression of colorectal cancer in recent research [46]. Particularly, LINC01106, under the regulation of m6A modification, promotes or suppresses cancer progression through its impact on specific signaling pathways. For example, LINC01106 can function as a sponge for miR-449b-5p, competitively binding and influencing the activity of miR-449b-5p, thus indirectly regulating VEGFA expression [47]. VEGFA is a crucial factor in angiogenesis, and alterations in its expression directly affect tumor vascular formation and growth [48]. Therefore, by investigating the role of LINC01106 in CRC, particularly how it affects tumor biological characteristics through m6A modification and the miR-449b-5p/VEGFA axis, new ideas and strategies for CRC treatment can be developed [47].

The primary objective of this study is to explore the role of m6A-modified LINC01106 in colorectal cancer, especially its regulation mechanism of YTHDF1 and its role in tumor angiogenesis through the miR-449b-5p-VEGFA signaling pathway. By analyzing the expression of LINC01106 and YTHDF1 in CRC and their association with clinical and pathological features, we aim to reveal their potential mechanisms in CRC development. Additionally, thorough validation of these findings through in vitro and in vivo experiments will deepen our understanding of the process of angiogenesis in CRC. The scientific and clinical significance of this study lies in the help it provides in developing novel therapeutic strategies targeting the m6A-modified LINC01106, offering more effective treatment choices for CRC patients. Furthermore, the study’s results may contribute to the prediction of prognosis in CRC patients, providing important information for clinical decision-making. In conclusion, this study will provide new ideas and evidence for molecular targeted therapy and personalized treatment for CRC (Table 1).

**Table 1:** The siRNA intrusion sequences of LINC01106, YTHDF1

Type	Number	Sequences	Length
siRNA	LINC01106 (human) siRNA-952 A	CCACAGAGUAUGAAGGUCATT	21
siRNA	LINC01106 (human) siRNA-640 A	CGGGAGAGGAGAAUGGAUUTT	21
siRNA	LINC01106 (human) siRNA-1184 A	GGAGGAAGGAGGCCACAAATT	21
siRNA	YTHDF1 (human) siRNA	ACGGCAGAGTCGAAACAAA	19

## Materials and methods

### Methods in bioinformatics

Firstly, the gene expression data of CRC and normal tissues obtained through the UALAN platform were subjected to standardization to eliminate systematic biases between different experimental batches. Next, differential expression analysis was carried out using statistical software packages to identify significantly differentially expressed genes. The correlation between YTHDF1, LINC01106, and VEGFA was assessed using Pearson or Spearman correlation analysis, and these associations were visually presented through heatmaps and scatter plots. Additionally, survival analysis of CRC patients was performed using clinical information from the TCGA database, including Kaplan-Meier survival curves, log-rank tests, and multivariable Cox regression analysis, to identify independent prognostic factors. Furthermore, bioinformatics tools were employed to predict the relationship between gene expression and patient survival rates, and the correlation between gene expression status and clinical characteristics of patients was evaluated.

### The Collection and ethical approval process of clinical tissue samples in colorectal cancer patients

This study was conducted at the Affiliated Hospital of Inner Mongolia Medical University and has been approved by the Institutional Review Board of the hospital, in accordance with the relevant provisions of the 1975 Helsinki Declaration (revised in 2013). All procedures involved in the study were reviewed and approved by the Human Research Ethics Committee of Inner Mongolia Medical University Affiliated Hospital (approval number: YKD202201235). From February 2019 to December 2022, we collected colorectal cancer (CRC) samples and adjacent non-tumor tissue samples from 30 CRC patients who underwent hysterectomy at the Department of Gastrointestinal Surgery at the Affiliated Hospital of Inner Mongolia Medical University. All patients participating in the study, or their relatives, have provided informed consent for the use of their tissue samples and related data for research purposes (Table 2).

**Table 2:** The primers used in this study

Gene	Sequence (5’-3’)
GAPDH	Forward primer: GTCTCCTCTGACTTCAACAGCG Reverse primer: ACCACCCTGTTGCTGTAGCCAA
LINC01106	Forward primer: TCCCCATATTCAAAAGCG Reverse primer: CATCAGAATGGCAAAGCA
YTHDF1	Forward primer: ACCTGTCCAGCTATTACCCG Reverse primer: TGGTGAGGTATGGAATCGGAG
VEGFA	Forward primer: CGCTCGGTGCTGGAATTTGAT Reverse primer: CCGTCGGCCCGATTCAAGT

### Cell culture and transfection experimental procedures

A variety of cell lines were utilized, including colorectal cancer (CRC) cell lines - HCT116, LOVO, SW480, DLD1, SW620 - normal colon epithelial cell line (NCM460), and human umbilical vein endothelial cells (HUVEC). All cell lines were obtained from Shanghai Yihua Biotech Co., Ltd., and verified to be free of mycoplasma contamination. The CRC cells were cultured in Dulbecco’s modified Eagle’s medium (DMEM) supplemented with 10% fetal bovine serum (FBS, PAN-Biotech, Cat#: P30-3306, Germany) and penicillin/streptomycin (100 U/mL, Wisent, Cat#: 450-201-EL, Canada), and maintained at 5% CO_2_ and 37 °C. HUVEC cells were cultured in specialized endothelial cell medium (ECM) (ScienCell, Cat#: 1001, USA), supplemented with 20% FBS (PAN-Biotech, Cat#: P30-3306, Germany).

For cell transfection experiments, Lipofectamine 2000 (Invitrogen, Carlsbad, California, USA) was used for siRNA transfection (siRNA final concentration of 50 nM), with the siRNA synthesized by Sango Biotech (China), and X-tremeGENE HP DNA transfection reagent (Roche, Mannheim, Germany) employed for plasmid DNA transfection (plasmid DNA final concentration of 1 µg/ml). During transfection, siRNA was diluted in 200 µl Opti-MEM (Thermo Fisher Scientific, Cat#. 31,985,070, USA) to achieve a final concentration of 50 nM, gently mixed by swirling 3–5 times, followed by gentle mixing of the transfection reagent according to the instructions, 4 µl Lipofectamine 2000 was diluted in 200 µl Opti-MEM, swirled gently 3–5 times, incubated at room temperature for 5 min; then the transfection reagent and siRNA dilution were mixed gently, swirled 3–5 times, left to stand at room temperature for 20 min; the transfection complex was then added to a 6-well plate ensuring a cell density of 50-70% for transfection. Following siRNA transfection for 6 h, fresh medium replacement was necessary, while plasmid transfection did not require medium change. After 48 h post-transfection, cells were collected for RNA and protein extraction. To validate the successful overexpression or knockdown of LINC01106, qPCR analysis was performed. In LOVO and SW480 cells, empty plasmid and pcDNA-LINC01106 overexpression plasmid were separately transfected; mRNA was extracted post-transfection, and LINC01106 expression levels were assessed via qPCR. For knockdown efficiency assessment, si-NC and si-LINC01106 #1, si-LINC01106 #2, si-LINC01106 #3 were each transfected into LOVO and SW480 cells separately, followed by mRNA extraction and qPCR analysis of LINC01106 expression levels.

### Construction of plasmids and interference sequences

To investigate the role of LINC01106, YTHDF1, and VEGFA genes in colorectal cancer, we designed and constructed specific siRNA interference sequences targeting these genes (Table S1). These siRNA sequences were designed to effectively reduce the expression levels of the target genes in cells. Additionally, we cloned the coding sequences (CDS) regions of human YTHDF1 (reference sequence: NM_017798.4), VEGFA (reference sequence: NM_001025366.3), and LINC01106 (reference sequence: NR_027244.1). These CDS regions were cloned into the pcDNA3.1 (+) vector (CROSE # V80020) for subsequent overexpression studies.

### DNA and RNA extraction and qRT-PCR

In order to analyze the expression of YTHDF1, LINC01106, and VEGFA genes, plasmid DNA containing these genes was initially extracted from Escherichia coli. This step was carried out using the NEB Monarch Plasmid Mini Kit II (Omega, GA, USA). Subsequently, total RNA was extracted from colorectal cancer (CRC) cell lines and fresh frozen clinical samples using Trizol reagent (Vazyme, Nanjing, China). The extracted RNA was utilized for cDNA synthesis, employing the PrimeScript RT Kit (Vazyme, Nanjing, China) according to the manufacturer’s protocol. The synthesized cDNA was stored at -20 °C for future use. For the extraction of RNA from the nucleus and cytoplasm, the Norgen Cytoplasmic & Nuclear RNA Purification Kit (Norgen Biotek, Cat#:21,000, Canada) was employed, with specific procedures outlined in the literature [49] and following the manufacturer’s instructions.

For quantitative analysis of the target gene expression levels, real-time quantitative PCR (qRT-PCR) experiments were conducted. These experiments were performed on the Step One Plus TM real-time PCR system (Applied Biosystems, Singapore), with an annealing temperature of 55 °C and 37 cycles of qPCR run each time. In qRT-PCR, GAPDH was utilized as the reference gene, and quantification was carried out using the 2^(-∆∆CT) method to ensure the accuracy and reliability of the experimental results. The sequences of the specific primers used are detailed in Table S2.

### Tube formation assay

To evaluate the impact of colorectal cancer (CRC) cells on angiogenesis, an tube formation assay was conducted. Initially, a pre-chilled 96-well plate was prepared and kept on ice throughout the experiment. Following that, 50 µl of thawed Corning gel, a matrix gel used to simulate the in vivo environment, was evenly distributed in each well. Subsequently, the transfected CRC cell supernatant was mixed with 3 × 10^4^ HUVEC cells and dropped into the solidified gel. This step aimed to mimic the microenvironment of angiogenesis. The mixed cells were then incubated for 6–8 h at 37 °C in a 5% CO_2_ incubator to allow for the formation of vascular structures. The process of angiogenesis was observed and captured at 4x magnification using an Olympus microscope IX53 (Olympus, Center Valley, Pennsylvania, USA). Employing Image Pro software, the number of formed vascular structures was quantified and analyzed. To ensure the reliability and repeatability of the experimental results, each experiment was repeated at least three times. Through this approach, the impact of CRC cells on angiogenic capacity could be quantitatively assessed, which is vital for understanding the angiogenic mechanism of colorectal cancer.

### Dual-luciferase reporter assay experiment

To investigate the targeted interaction between LINC01106 and miR-449b-5p, a dual-luciferase reporter system was employed in the present study. The wild-type (WT) or mutant sequences of LINC01106 were inserted into the psiCHECK-2 plasmid (Promega, Cat#: C8021, USA). Subsequently, cells were co-transfected with the luciferase reporter plasmid and miR-449b-5p mimic. The cells used were LOVO or SW480. After 48 h, 5 × 10^5^ cells were harvested for luciferase activity measurement. The relative enzyme activity was determined using the Dual-Luciferase® Reporter Assay System (Promega, Cat#: E1910, USA) following the manufacturer’s instructions. The firefly luciferase activity was normalized to Renilla luciferase activity, and each sample group had three biological replicates.

Similarly, to investigate the targeted regulatory effect of miR-449b-5p on VEGFA, the WT or mutant sequences of VEGFA-3’UTR were constructed into the psiCHECK-2 plasmid. The subsequent procedures were analogous to those described above for LINC01106 and miR-449b-5p interaction analysis. After 48 h, 5 × 10^5^ cells were collected for luciferase activity measurement using the Dual-Luciferase® Reporter Assay System. The firefly luciferase activity was normalized to Renilla luciferase activity, and each sample group was conducted in triplicate for statistical reliability.

### Western blot

Western blot (WB) technique was used in this study to detect the protein expression levels of YTHDF1 and VEGFA. Initially, cell samples were collected and lysed to extract the proteins, and their concentrations were quantified using appropriate methods. Subsequently, the extracted protein samples were loaded onto a 10% SDS-PAGE gel for electrophoretic separation. After completion of electrophoresis, the proteins were transferred from the gel to a polyvinylidene fluoride (PVDF) membrane activated with methanol. The transferred membrane was then incubated in a blocking solution containing 5% milk at a temperature of 20 ± 5 °C for 1 h to prevent non-specific binding. Following this, the membrane was incubated overnight at 4 °C with specific primary antibodies. The primary antibodies used included anti-YTHDF1 antibody (1:1000, 17479-1-AP, ProteinTech, China), anti-VEGFA antibody (1:2000, AB185238, Abcam, USA), and GAPDH antibody as an internal control (1:1000, 60004-1-Ig, ProteinTech, China). After incubation with the primary antibody, the membrane was washed and incubated with respective secondary antibodies, including goat anti-rabbit and goat anti-mouse antibodies, diluted at a ratio of 1:5000, at room temperature for 1 h. Finally, the protein signals on the membrane were detected using a chemiluminescent gel imaging system (Tanon 5200, Shanghai, China).

### Immunohistochemistry (IHC)

Initially, tissue samples were fixed and cut into 4 μm thick sections. The first step in section processing involved treatment with ethylenediaminetetraacetic acid (EDTA) as an antigen retrieval solution to facilitate antibody binding. Subsequently, the sections were incubated with the following antibodies: CD31 antibody (1:2000, AB76533, Abcam, Cambridge, MA, USA) for labeling of endothelial cells, VEGFA antibody (1:400, AB185238, Abcam, Cambridge, USA) for detection of VEGFA protein, m6A antibody (1:200, AB151230, Abcam, Cambridge, MA, USA) for detection of m6A modification, and YTHDF1 antibody (1:500, AB195352, Abcam, Cambridge, MA, USA) for detection of YTHDF1 protein. These sections were incubated with primary antibodies overnight at 4 °C to ensure optimal antibody binding. After incubation, the sections were washed and incubated with respective secondary antibodies at 37 °C for 1 h. Finally, the sections were stained and imaged to visualize and evaluate the expression and localization of the target proteins. The calculation method for the IHC score is as follows: IHC score = Cell staining intensity score x Percentage of positive cells score. Cell staining intensity score is graded into 4 levels: negative, scored as 0; weak positive, scored as 1; positive, scored as 2; strong positive, scored as 3. The percentage of positive cells score is divided into 4 levels: 0%≤ positive cell percentage ≤ 25%, scored as 1; 25%< positive cell percentage ≤ 50%, scored as 2; 50%< positive cell percentage ≤ 75%, scored as 3; 75%< positive cell percentage ≤ 100%, scored as 4.

### m6A-qRT-PCR

First, total RNA was extracted from CRC cell lines using Trizol reagent (Invitrogen, Carlsbad, California, USA). The extracted RNA ($\sim$100 µg) was treated with DNase (Takara, Shiga, Japan) in a 150 µl reaction system to remove DNA contamination. Following DNase treatment, total RNA was extracted again using Trizol reagent. Next, RNA fragmentation was carried out at 71 °C for 5 min using RNA fragmentation reagent (Invitrogen, Carlsbad, California, USA) to generate RNA fragments suitable for immunoprecipitation. The fragmented RNA was immediately mixed with stop buffer and then re-extracted with Trizol reagent. The extracted RNA fragments were dissolved in 200 µl of DEPC water. Approximately 160 µl of RNA fragments were diluted in MeRIP buffer (containing 150 mM potassium chloride, 25 mM Tris, 5 mM EDTA, 0.5% Triton X-100, 0.5 mM DTT, proteinase inhibitor (1:100, Invitrogen, Carlsbad, USA), and RNase inhibitor (1:1000, Wuhan Clone, China)). The diluted samples were divided into two parts, one part was treated with anti-m6A antibody (antibody, Wuhan), and the other part was treated with control IgG antibody. Both parts were incubated with protein A/G magnetic beads (MCE, Monmouth Junction, USA) in 900 µl of RNA Immunoprecipitation (RIP) lysis buffer at 4 °C for 4 h. After incubation, 20 µl of RNA fragments were collected and subjected to immunoprecipitation. The beads were washed four times with RIP buffer and then treated with 130 µl of MeRIP buffer containing 10 µl 10% SDS and 10 µl proteinase K (Takara, Shiga, Japan) at 55 °C for 30 min. The treated liquid was transferred to a new tube and phase separation was performed by adding 1 ml of Trizol reagent and chloroform. After centrifugation, the upper aqueous phase was collected and mixed with 1/10 volume of 3 M sodium acetate and an equal volume of isopropanol, as well as glycogen (final concentration 100 µg/ml). The samples were stored overnight at -80 °C and then centrifuged at 12,000×g, 4 °C for 15 min. The pellet was washed twice with 75% ethanol and finally dissolved in DEPC water. Two-step quantitative RT-PCR analysis was performed using Takara RT-PCR kits (Takara, Shiga, Japan).

RNA-binding protein immunoprecipitation (RIP) First, CRC cells were washed twice with ice-cold phosphate-buffered saline (PBS), followed by treatment with 1 mL of RIP lysis buffer (containing 150 mM potassium chloride, 25 mM Tris, 5 mM EDTA, 0.5% Triton X-100, 0.5 mM DTT, 1:100 protease inhibitor, and 1:1000 RNase inhibitor) for 30 min to lyse the cells and release RNA and its associated proteins. Subsequently, the cell lysate was centrifuged at 12,000×g, 4 °C for 15 min to remove cellular debris. 10% of the supernatant was collected as the input sample, while the remaining supernatant was incubated with the following antibodies: anti-METTL3 (Ab195352, Abcam, USA), anti-METTL14 (Ab195408, Abcam, USA), anti-FTO (MERCK 252, USA), and an IgG control antibody (MCE, USA). These samples were incubated with protein A/G magnetic beads (MCE, USA) in 900 µL of RIP lysis buffer at 4 °C for 4 h to allow the binding of RNA to specific proteins. After incubation, the beads were subjected to immunoprecipitation and washed four times with RIP buffer. Subsequently, the beads were treated with 130 µL of MeRIP buffer containing 10 µL 10% SDS and 10 µL proteinase K (Takara, Shiga, Japan) at 55 °C for 30 min. Following treatment, immunoprecipitation (IP) or input group RNA was recovered using Trizol reagent (Invitrogen, Carlsbad, California, USA) according to the manufacturer’s instructions and analyzed by quantitative RT-PCR. The enrichment rate of RNA was calculated as the ratio of its quantity in IP to the input quantity, thereby evaluating the interaction between specific RNA-binding proteins and target RNA.

### Nude mouse matrix gel plug test

In this study, we conducted the nude mouse matrix gel plug test to evaluate the angiogenic ability of colorectal cancer (CRC) cells in vivo. The experiment was approved by the Institutional Animal Care and Use Committee of Nanjing Medical University (IACUC-1,705,037), and all animal care and procedures followed the standards set by the Experimental Animal Management Regulations approved by the State Council of the People’s Republic of China.

8-week-old female BALB/c nude mice, obtained from the Vivofrederick Animal Resources Centre and raised under specific pathogen-free conditions, were used in the experiment. Prior to the experiment, CRC cells were resuspended in serum-free culture medium. Subsequently, a mixture of 0.2 ml of the cell suspension and 0.2 ml of high-concentration extracellular matrix gel (BD Biosciences, CA) was immediately injected into the backs of the nude mice, with each experimental group consisting of 5 mice. After 14 days post-injection, the mice were euthanized, and the matrix thrombi were extracted. Photographs were taken at 4x magnification using an Olympus stereomicroscope MVX10 (Olympus, Center Valley, Pennsylvania, USA). For microvessel perfusion, a total of 200 mL FITC-conjugated lectin (1 mg/ml) was intravenously injected in the last 30 min before mouse death. Blood hemoglobin was analyzed using the Drabkin reagent kit (Sigma-Aldrich) according to the manufacturer’s instructions. The final hemoglobin concentration was measured at 540 nm, and calculations were performed based on a standard calibration curve. Finally, immunohistochemistry was used to assess the expression levels of HE, CD31, and VEGFa for evaluating angiogenesis.

### RNA stability analysis

To assess the stability of specific RNAs in colorectal cancer (CRC) cells, we conducted an RNA stability analysis. Firstly, CRC cells were seeded in a 6-well plate overnight to ensure proper cell adhesion and growth. Subsequently, cells were treated with actinomycin D (5 µg/mL, HY-17,559, MedChemExpress) and cell samples were collected at 0, 3, 6, and 9 h afterwards. This step aimed to inhibit new RNA synthesis, allowing us to observe the degradation process of existing RNA molecules. Next, total RNA was isolated from the treated cells using Trizol reagent (Invitrogen, USA). Then, the expression levels of specific mRNAs were analyzed using quantitative real-time reverse transcription polymerase chain reaction (qRT-PCR). To accurately assess RNA stability, the mRNA expression levels at each time point were normalized to GAPDH (the internal control gene). Finally, the half-life of the mRNA was estimated through linear regression analysis. This analysis was based on the changes in mRNA expression levels at different time points, enabling the calculation of RNA degradation rate and half-life period.

### Statistical analysis

All statistical analyses in this study were performed using GraphPad Prism 7 software to ensure accurate data processing and clear visualization. Experimental results were presented as mean ± standard deviation (SD) to provide an intuitive representation of the central tendency and dispersion of the data. Based on the characteristics of the data, we selected two-tailed Student’s t-test, two-tailed paired t-test to compare the differences between different experimental groups, with a significance level of *P* < 0.05 considered statistically significant. To facilitate the presentation of significant differences, we used different levels of labeling such as *P* < 0.05, *P* < 0.01, *P* < 0.001, **P < 0.0001. Additionally, Image J software was used for quantitative analysis of image data, enhancing the accuracy of the results. Each experiment was performed with at least three biological replicates to improve the reliability and reproducibility of the results.

## Results

### Expression of YTHDF1, LINC01106, and VEGFA and their correlation with overall survival in colorectal cancer (CRC) patients

In the pathogenesis of colorectal cancer (CRC), the regulation and interaction of gene expression play crucial roles. According to the literature, LINC01106 is associated with the proliferation, migration, and invasion phenotypes of various tumor cells [47, 50]. Additionally, LINC01106 can undergo m6A modification [51]. However, there have been no reports on the relationship between LINC01106 and m6A readers that regulate lncRNAs (such as YTHDF1), as well as the regulation of VEGFA that promotes tumor angiogenesis. This study aims to investigate the expression patterns of YTHDF1, LINC01106, and VEGFA in CRC, as well as their potential associations with overall patient survival rates.

**Fig. 1:**
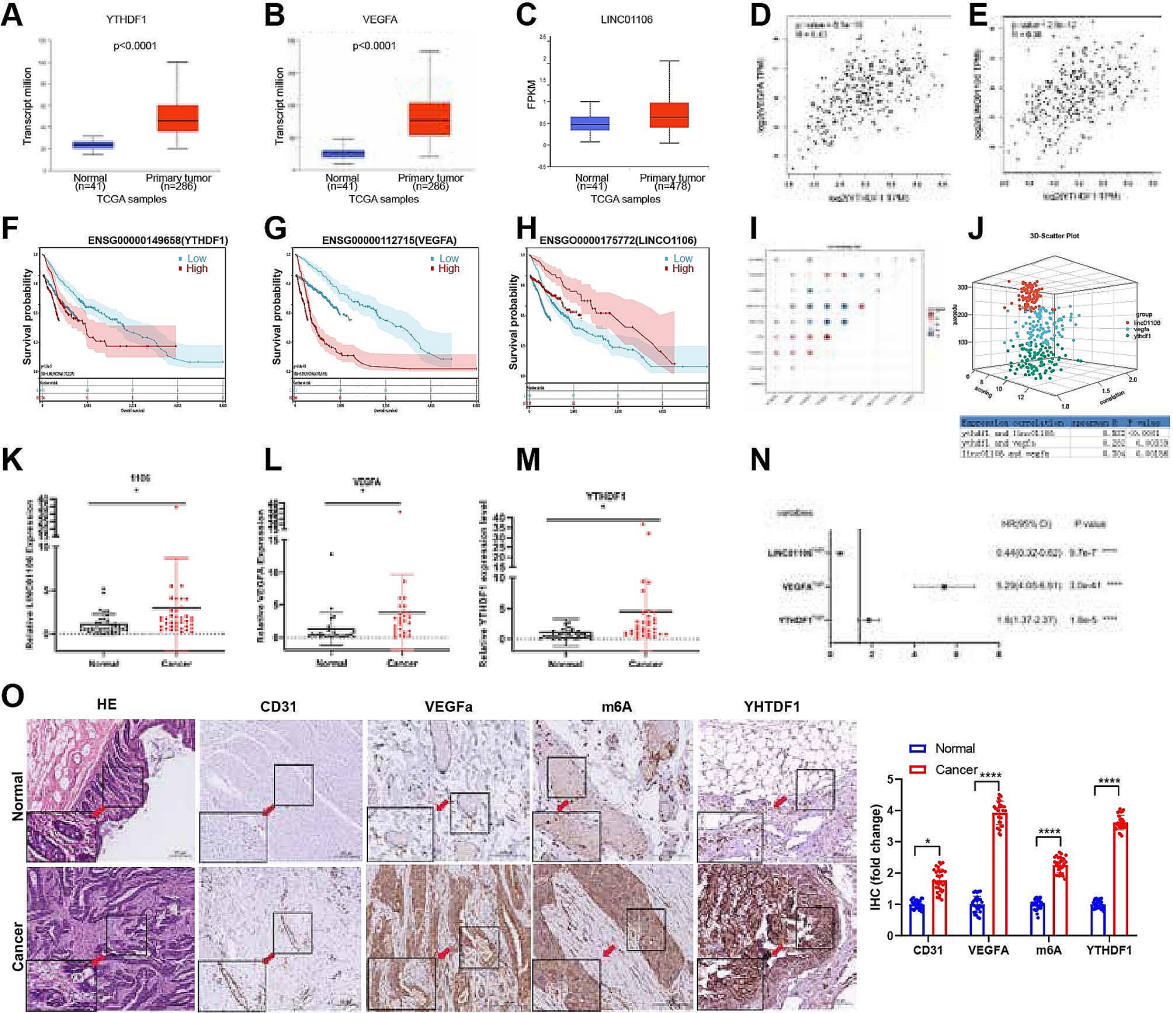
Correlation analysis of expression levels of YTHDF1, LINC01106, and VEGFA in colorectal cancer and patient prognosis. Note: (**A**) Expression levels of YTHDF1 and VEGFA in colorectal cancer (COAD) samples from TCGA database. (**B**) Expression levels of YTHDF1 and VEGFA in colorectal cancer (COAD) samples from TCGA database. (**C**) Expression level of LINC01106 in colorectal cancer (COAD) samples from GEPIA database. (**D**) Positive correlation analysis of YTHDF1 expression with LINC01106 in colorectal cancer. (**E**) Positive correlation analysis of YTHDF1 expression with VEGFA in colorectal cancer. (**F**-**H**) Correlation analysis between expression levels of YTHDF1, VEGFA, and LINC01106 and overall survival rate in colorectal cancer patients. (I) Correlation matrix analysis between m6A gene expression and hypoxia-induced autophagy in colorectal cancer tissue. (**J**) Three-dimensional scatter plot representing YTHDF1, LINC01106, and VEGFA in colorectal cancer (CRC) tumor tissue. (**K**-**M**) Expression level analysis of YTHDF1, LINC01106, and VEGFA in colorectal cancer tissue. (**N**) Multivariate Cox regression analysis of overall survival in colorectal cancer patients. (**O**) Immunohistochemical staining analysis of m6A antibody, YTHDF1, LINC01106, and VEGFA in colorectal cancer tissue. The bar graph represents the quantitative analysis results of the change multiples in IHC positive rates. The IHC scores were calculated based on images of 30 pairs of samples, normalized against the control group’s IHC score, to determine the change multiples in IHC scores for each experimental group relative to the control group. Error bars represent mean ± standard error (SEM), dots represent values from each experiment; For Fig. 1A, B, C, K, L, M, Student’s t-test was used to calculated significance and* *P* < 0.05; ** *P* < 0.01, *** *P* < 0.001

Through analysis of data from the UALCAN platform, we observed significantly higher expression levels of YTHDF1, LINC01106, and VEGFA in CRC samples compared to normal tissues (Fig. 1A-C). Further correlation analysis revealed significant positive associations between YTHDF1 and LINC01106 as well as VEGFA (Fig. 1D-E). Analysis of the Cancer Genome Atlas (TCGA) CRC database showed that the high expression of YTHDF1, LINC01106, and VEGFA was closely associated with poorer overall survival (Fig. 1F-H). Additionally, m6A gene expression analysis suggested that YTHDF1 may play an important role in CRC angiogenesis (Table 3), and a three-dimensional scatter plot was used to visually demonstrate the relationship between YTHDF1 and LINC01106 as well as VEGFA (Fig. 1I-J).

**Table 3:** The expression analysis of m6A-related genes revealed the association of YTHDF1 and CRC angiogenesis

Gene	M1	M2	M3	M4	M5	M6	M7	M8	M9	M10
YTHDC1	0.9	-0.0178	0.15	0.3	-0.351	-0.165	-0.461	0.16	0.326	-0.241
YTHDF2	0.15	0.67	0.1	0.317	0.219	-0.0655	-0.107	0.004338	0.276	0.28
YTHDF3	-0.0178	0.1	0.79	0.328	0.1	-0.14	0.0163	0.0861	0.182	-0.134
YTHDF1	0.3	0.317	0.328	0.68	0.0431	-0.298	-0.248	0.283	0.578	0.101
YTHDC2	-0.351	0.219	0.1	0.0431	0.74	0.0429	0.166	0.246	0.11	0.094
METTL3	-0.165	-0.0655	-0.14	-0.298	0.0429	0.78	0.297	-0.0137	-0.49	0.005023
METTL14	-0.461	-0.107	0.0136	-0.248	0.166	0.297	0.86	-0.19	-0.618	0.088
RBM15	0.16	0.275	-0.0861	0.283	0.246	-0.0137	-0.19	0.6	0.198	0.0454
FTO	0.326	0.276	0.182	0.578	0.11	-0.49	-0.618	0.198	0.84	0.105
ALKBH5	-0.241	0.28	-0.134	0.101	0.094	0.005023	0.0888	0.0454	0.105	0.75

Analysis of data from the UALAN platform revealed that the expression levels of YTHDF1, LINC01106, and VEGFA in CRC samples (*n* = 286, patient samples) were significantly higher than those in normal tissues (*n* = 41, samples from healthy individuals) (Fig. 1A-C). Further correlation analysis of expression levels demonstrated a significant positive correlation between YTHDF1 and both LINC01106 and VEGFA. The correlation coefficient between the expression levels of YTHDF1 and VEGFA in CRC samples was 0.43, with a p-value 8.9e-16. Additionally, the correlation coefficient between the expression levels of YTHDF1 and VEGFA in CRC samples was 0.38, with a p-value of 2.3e-12 (Fig. 1D-E). Analysis of The Cancer Genome Atlas (TCGA) CRC database revealed that the high expression of YTHDF1, LINC01106, and VEGFA was closely associated with poorer overall survival rates (Fig. 1F-H). Furthermore, analysis of the expression levels of m6A modification-related genes in CRC tissues from patients M1-M10 indicated a strong correlation between YTHDF1 and CRC angiogenesis, suggesting a potential crucial role of YTHDF1 in CRC angiogenesis (Table 3). This relationship between YTHDF1, LINC01106, and VEGFA was visually represented through a three-dimensional scatter plot (Fig. 1I-J).

In a comparison of 30 pairs of CRC and normal tissue samples, we found significant upregulation of YTHDF1, LINC01106, and VEGFA in CRC tissues with statistical significance (Fig. 1K-M). Further analysis of the TCGA database indicated that the expression of LINC01106 was significantly elevated in CRC patients with different stages and metastatic status (Supplementary Fig. 1A-D). Multivariate Cox regression analysis confirmed LINC01106 as an independent prognostic factor in CRC patients (HR 4.1, 95% CI 3.4–5.6, *P* = 0.054) (Fig. 1N). Immunohistochemistry (IHC) experiments further confirmed the significant upregulation of CD31 and VEGFA protein levels associated with angiogenesis in CRC, as well as the upregulation of m6A and m6A recognition protein YTHDF1, suggesting the potential role of m6A RNA modification in CRC angiogenesis (Fig. 1O).

In conclusion, the high expression of YTHDF1, LINC01106, and VEGFA is closely correlated with poorer overall survival in colorectal cancer, particularly the identification of LINC01106 as an independent prognostic factor, providing new insights into the molecular mechanisms and potential therapeutic targets of CRC. According to the literature, it has been reported that LINC01106 drives the growth and stemness of colorectal cancer cells through positive feedback regulation of Gli family factors [47]. Our findings align with the reported phenotype, and we have identified novel genes involved in the regulation of CRC development by LINC01106.

### Regulation of angiogenesis by LINC01106 in colorectal cancer

In the process of colorectal cancer (CRC) development, angiogenesis plays a crucial role. Our study focuses on elucidating the role of long non-coding RNA LINC01106 in CRC angiogenesis and its potential mechanisms. Through a series of in vitro and in vivo experiments conducted in CRC cell lines, we aimed to uncover the impact of LINC01106 on angiogenesis and its possible regulatory mechanisms.

**Fig. 2:**
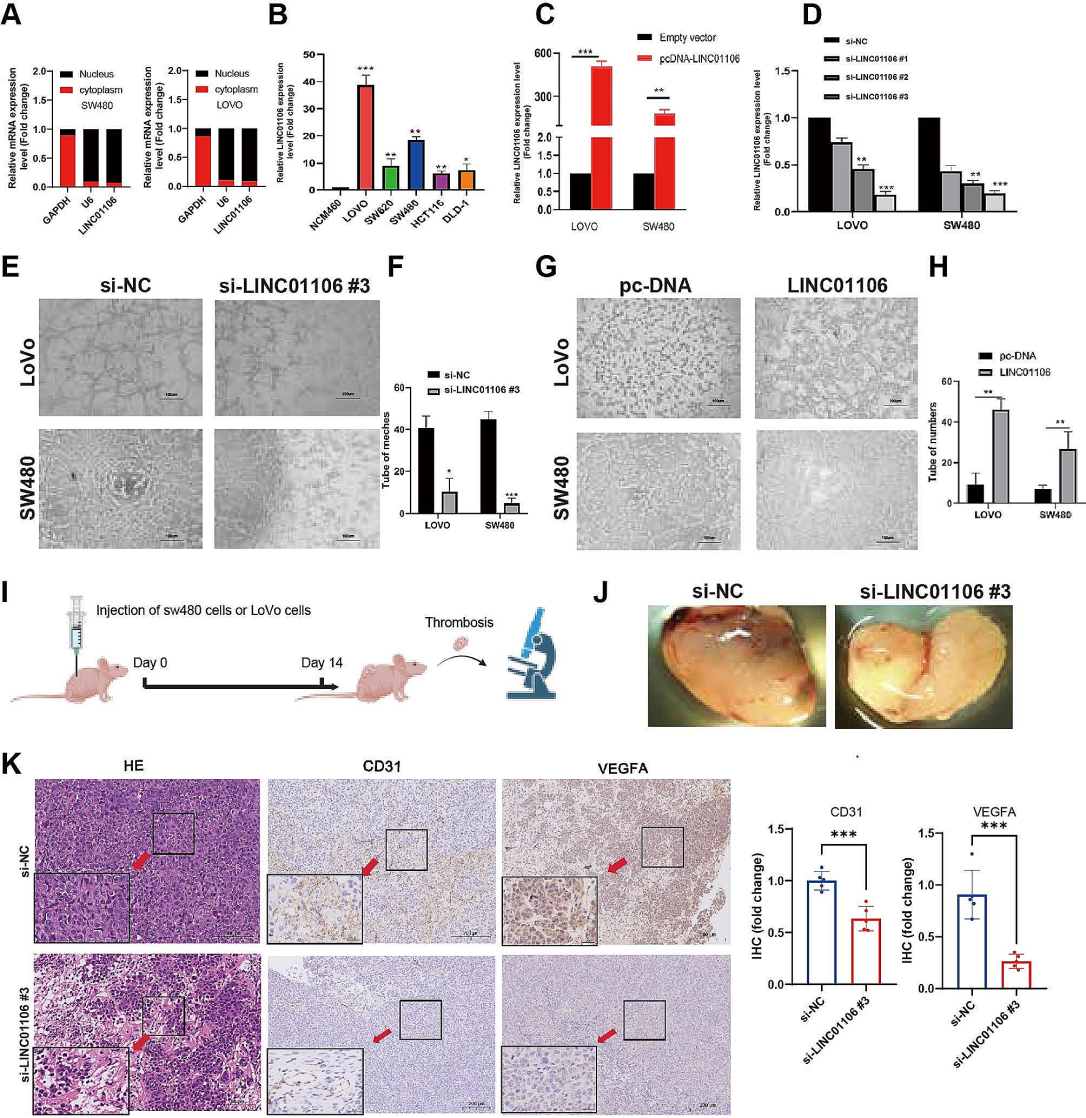
Experimental validation of the regulatory role of LINC01106 in colorectal cancer cell angiogenesis. Note: (**A**) The expression levels of LINC01106 in the nuclei (red) and cytoplasm (black) of LOVO and SW480 cells assessed using RT-qPCR, with GAPDH and U6 serving as positive controls for nuclear-cytoplasmic separation. (**B**) The relative expression of LINC01106 in different CRC cell lines was presented; expression levels were normalized to the expression of the internal control gene GAPDH in normal human colonic epithelial cells and all CRC cell lines, further normalizing to the expression level in normal colonic epithelial cells to obtain fold changes in LINC01106 expression levels in different CRC cell lines relative to normal colonic cells. (**C**) Overexpression of LINC01106 was performed in LOVO and SW480 cells, with black denoting empty vector control and red denoting the overexpression vector of LINC01106. (**D**) Silencing of LINC01106 in LOVO and SW480 cells was achieved using three different siRNA sequences to validate its regulatory effect. (**E**-**H**) The impact of LINC01106 silencing and overexpression on the angiogenic capability of CRC cells was evaluated through tubule formation assays. (**I**) The establishment process of BALB/c-nu mice subcutaneous xenograft CRC model is described. (**J**) Assessment of subcutaneous angiogenesis in BALB/c-nu mice comparing the control group to the si-LINC01106 group. (**K**) Results of HE staining in blood vessels after silencing LINC01106 and immunohistochemical staining for CD31/VEGFA to validate the impact of LINC01106 on angiogenesis. The bar graph displays the quantitative analysis results of the fold change in IHC scores. IHC scores were calculated using images of 30 sample pairs and then normalized to the control group to determine the fold change in IHC scores for each experimental group relative to the control group. For Fig. 2B, C, D, F, H, Student’s t-test was used to calculated significance and statistical significance is indicated as * *P* < 0.05; ** *P* < 0.01, *** *P* < 0.001 to represent significant differences between different treatment groups

Firstly, using RT-qPCR, we assessed the expression levels of LINC01106 in the nucleus and cytoplasm of LOVO and SW480 cells, using GAPDH and U6 as nuclear-cytoplasmic positive controls (Fig. 2A). Subsequently, we utilized qPCR to examine the expression of LINC01106 in normal human colonic epithelial cells (NCM-460) and various CRC cell lines (Fig. 2B). Our findings revealed a significant upregulation of LINC01106 expression in CRC cells compared to normal human colonic epithelial cells (NCM-460), with particularly higher expression levels observed in the LOVO and SW480 cell lines. Following this, we conducted overexpression and silencing experiments on LINC01106 in LOVO and SW480 cells using three different siRNA sequences, selecting the most effective sequence for subsequent experiments (Fig. 2C-D). To evaluate the influence of LINC01106 on the angiogenic capacity of CRC cells, we performed tube formation assays (Fig. 2E-H). Furthermore, we conducted in vivo experiments using a subcutaneous xenograft CRC model in BALB/c-nu mice to further investigate the role of LINC01106 (Fig. 2I-J). Finally, we confirmed the impact of LINC01106 on angiogenesis in vivo by performing HE staining and immunohistochemistry for CD34/VEGFa on the LINC01106-silenced xenografts (Fig. 2K).

In summary, our study demonstrates that LINC01106 is highly expressed in CRC cells and its silencing significantly inhibits the angiogenic capacity of CRC cells.

### YTHDF1 regulates the expression of LINC01106 in colorectal cancer through m6A modification

Numerous studies in the literature have explored the association of LINC01106 with tumor cell proliferation, such as its role in driving the growth and stemness of colorectal cancer cells through positive feedback regulation of Gli family factors [47]. Additionally, reports indicate that LINC01106 promotes bladder cancer progression [51], enhances proliferation in colorectal cancer cells by activating the STAT3 pathway [52], while its knockdown inhibits gastric cancer cell proliferation [53] and restrains proliferation, migration, and invasion in endometrial cancer [50]. Consistent with these findings, our previous research demonstrated LINC01106’s promotion of angiogenic capabilities in colorectal cancer cells in vitro and in vivo. Furthermore, through literature research, it was discovered that LINC01106 undergoes m6A modification, thereby enhancing its stability [51]. The m6A modification on lncRNAs provides binding sites for m6A readers, inducing the binding of RNA-binding proteins (RBPs) to regulate the functionality of LINC01106 [54]. YTHDF1, identified as a highly expressed m6A reader in CRC, has drawn our attention due to the reduced expression levels of the extensively studied YTHDC1 in CRC, prompting a focus on the regulatory relationship between YTHDF1 and LINC01106.

**Fig. 3:**
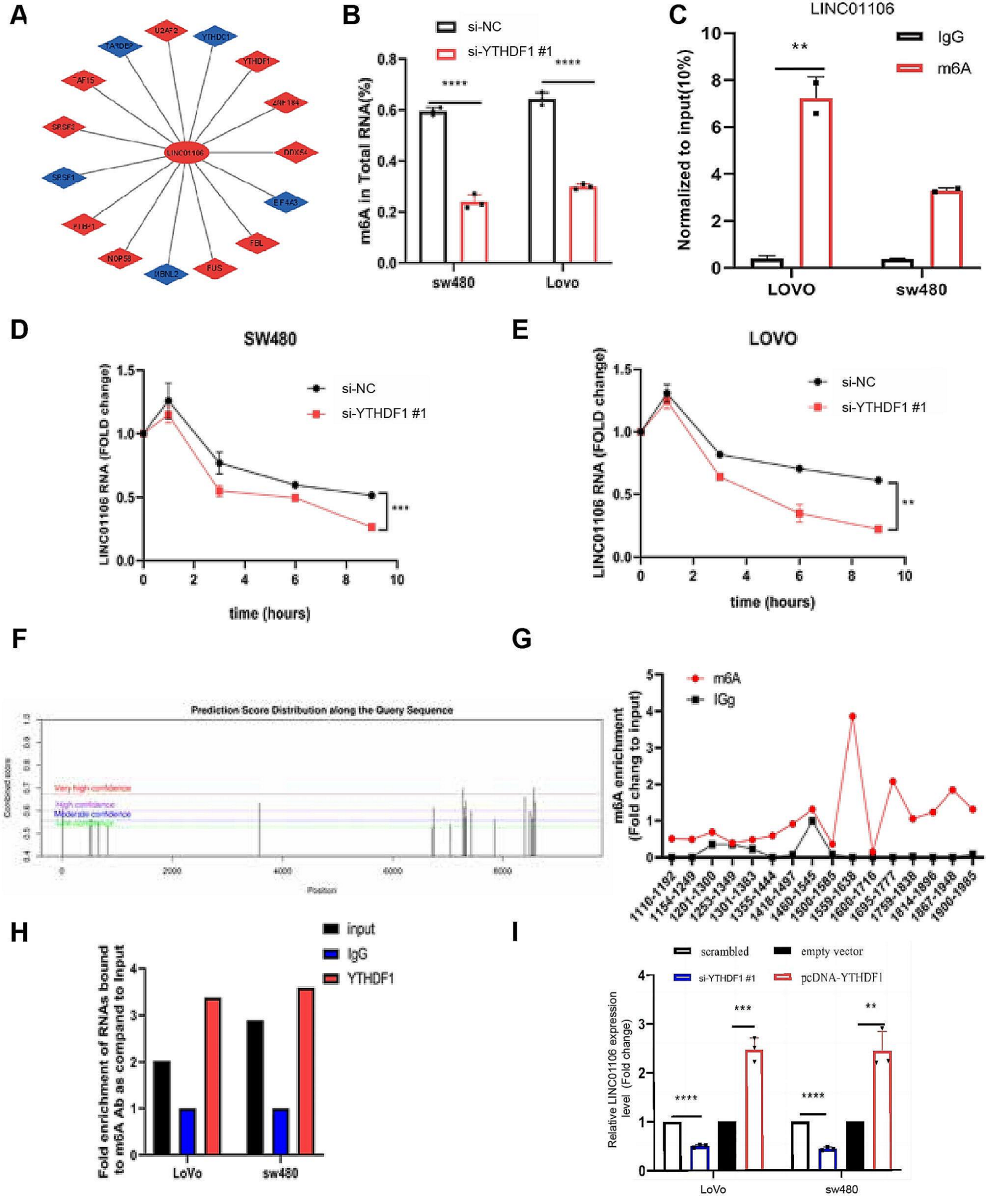
Study on m6A modification of LINC01106 and its interaction with YTHDF1 in colorectal cancer cells. Note: (**A**) The interaction network between LINC01106 and various RNA-binding proteins (RBPs) based on analysis of CLIP-seq data. The central red ellipse represents LINC01106, the surrounding blue rhombuses represent RBPs known to directly interact with LINC01106, while the red rhombuses represent RBPs that may regulate LINC01106 function through other indirect mechanisms. The lines indicate the interaction relationships between LINC01106 and the RBPs; (**B**) RIP-qPCR analysis of m6A immunoprecipitation in SW480 and LOVO cells shows enrichment of m6A in these CRC cells, especially in LINC01106. (**C**-**E**) qRT-PCR to assess the expression levels of LINC01106 at different time points after YTHDF1 knockdown and evaluate the impact of YTHDF1 on LINC01106 expression. (**F**) Prediction of m6A modification sites on LINC01106 using the SRAMP database for further analysis. (**G**) Validation of m6A modification enrichment in LINC01106 RNA through m6A immunoprecipitation-PCR (MeRIP) experiments. (**H**) Investigation of the binding between YTHDF1 and LINC01106 in Lovo and SW480 cells through YTHDF1 immunoprecipitation (RIP) experiments. (1) RT-qPCR analysis confirms decreased expression of LINC01106 in YTHDF1 knockout cells and increased expression in YTHDF1 overexpressing cells, further confirming the regulatory role of YTHDF1 on LINC01106 expression. For Fig. 3B, C, D, E, Student’s t-test was used to calculated significance and for Fig. 3I, one-way ANOVA was performed. Statistical significance is indicated as * *P* < 0.05; ** *P* < 0.01, *** *P* < 0.001 to represent significant differences between different treatment groups

To delve deeper into understanding the mechanistic role of YTHDF1 in colorectal cancer (CRC) progression, this study initially conducted CLIP-seq data analysis to investigate the interactions between RNA-binding proteins (RBPs) and RNA (Fig. 3A; Table 4), with a specific emphasis on YTHDF1. Specifically, we focused on the interactions between YTHDF1 and YTHDC1, two RBPs, and LINC01106. We observed that YTHDC1 was downregulated in CRC, leading us to choose YTHDF1 as the focal point of our study. Initially, we utilized an m6A methylated quantification kit to examine the impact of YTHDF1 on the overall m6A levels in Lovo and SW480 cells. The results demonstrated a significant decrease in m6A levels in cells with reduced YTHDF1 expression (Fig. 3B). Additionally, MeRIP-qPCR analysis revealed significant m6A enrichment in LINC01106 mRNA in both SW480 and Lovo cells (Fig. 3C). Silencing YTHDF1 further enhanced the efficiency of LINC01106 silencing over time (1–9 h), regardless of cell line (SW480 or LOVO) (Fig. 3D-E). Predictive analysis of LINC01106 transcripts using the SRAMP database identified multiple m6A motifs within the sequence (Fig. 3F). MeRIP experiments further confirmed a high m6A binding site at the 3’UTR end of LINC01106 (Fig. 3G). RIP experiments revealed the interaction between YTHDF1 and LINC01106 (Fig. 3H), and subsequent knockdown of YTHDF1 resulted in a decreased mRNA level of LINC01106 (Fig. 3I). Taken together, our study elucidates the importance of YTHDF1 in the regulation of LINC01106 expression through m6A-dependent mechanisms, thereby playing a significant role in the development of colorectal cancer.

**Table 4:** Dynamic regulation of RBP-RNA interaction sites in colorectal cancer

RBP	geneID	geneName	geneType	clusterNum	clipExpNum	clipIDnum	HepG2	K562	pancancerNum
DDX54	ENSG00000175772	LINC01106	lincRNA	1	1	1	NA	NA	13
EIF4A3	ENSG00000175772	LINC01106	lincRNA	1	1	1	NA	NA	11
FBL	ENSG00000175772	LINC01106	lincRNA	1	1	1	NA	NA	13
FUS	ENSG00000175772	LINC01106	lincRNA	17	3	19	NA	NA	15
MBNL2	ENSG00000175772	LINC01106	lincRNA	3	2	3	NA	NA	19
NOP58	ENSG00000175772	LINC01106	lincRNA	1	2	2	NA	NA	24
PTBP1	ENSG00000175772	LINC01106	lincRNA	2	2	3	1.666	NA	12
SRSF1	ENSG00000175772	LINC01106	lincRNA	4	2	4	NA	NA	22
SRSF3	ENSG00000175772	LINC01106	lincRNA	2	1	2	NA	NA	21
TAF15	ENSG00000175772	LINC01106	lincRNA	20	5	33	NA	NA	17
TARDBP	ENSG00000175772	LINC01106	lincRNA	2	2	2	NA	NA	20
U2AF2	ENSG00000175772	LINC01106	lincRNA	2	1	2	NA	NA	16
YTHDC1	ENSG00000175772	LINC01106	lincRNA	5	3	6	NA	NA	24
YTHDF1	ENSG00000175772	LINC01106	lincRNA	2	2	3	NA	NA	17
ZNF184	ENSG00000175772	LINC01106	lincRNA	2	1	2	NA	NA	18

### Role of YTHDF1 in angiogenesis in colorectal cancer

To gain a deeper understanding of the role of YTHDF1 in angiogenesis in colorectal cancer (CRC), we conducted a series of experiments in multiple CRC cell lines, with a focus on YTHDF1 expression patterns and their impact on angiogenesis.

**Fig. 4:**
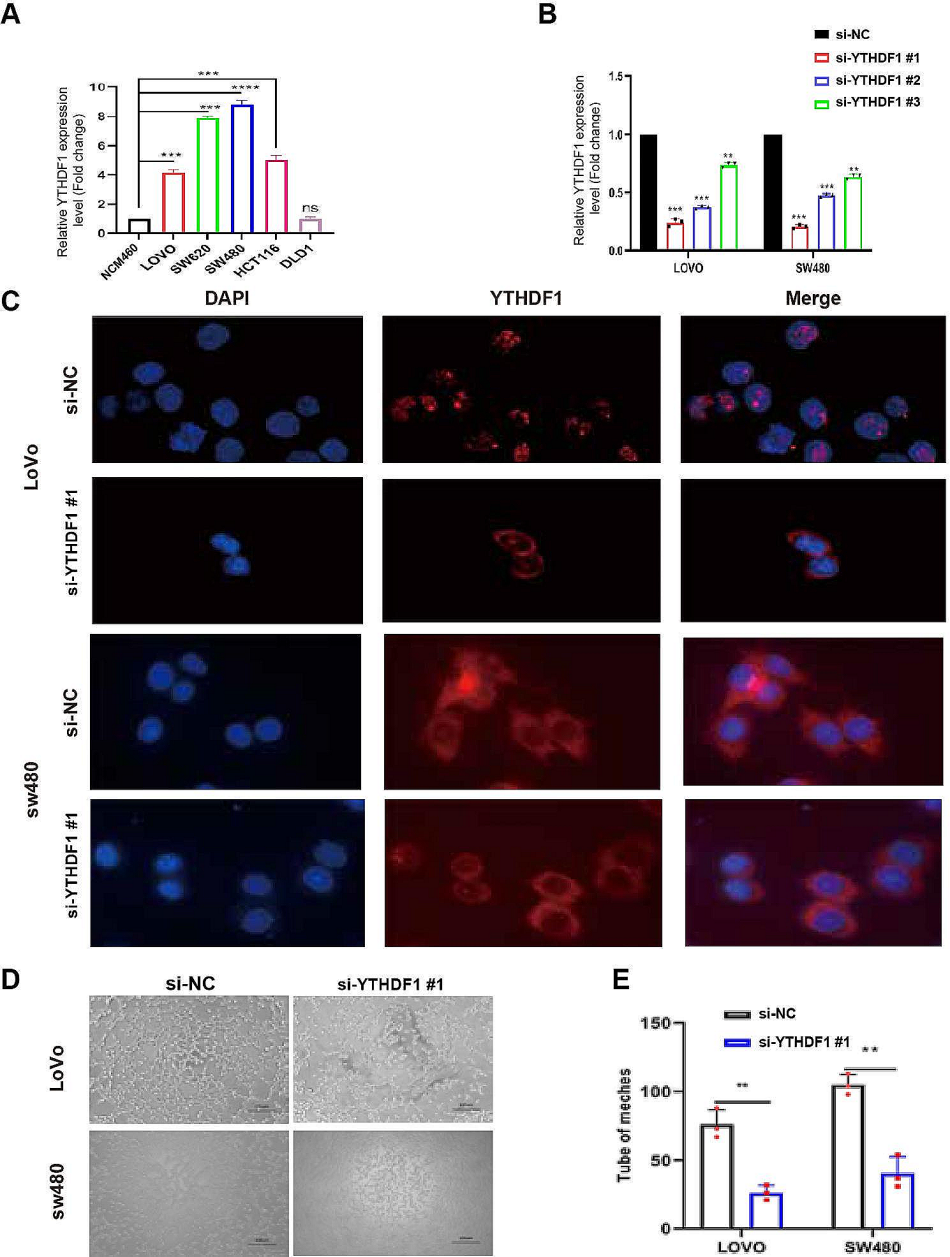
The role of YTHDF1 in promoting angiogenesis in colorectal cancer cells. Note: (**A**) Using qRT-PCR to verify the expression levels of YTHDF1 in different colorectal cancer (CRC) cell lines lays the foundation for establishing experimental models. The y-axis represents fold change, with the expression of the NCM460 group set as 1. (**B**) Employing qRT-PCR to evaluate the silencing efficiency of the YTHDF1-targeting plasmid in LOVO and SW480 cells provides a basis for subsequent experiments, with the y-axis representing fold change and the expression of si-NC group set as 1. (**C**) YTHDF1 expression was observed to decrease in LoVo and SW480 cells after YTHDF1 knockdown using immunofluorescence (IF) technique, revealing changes in YTHDF1 localization and its cellular functions (scale bar: 100 μm). (**D**-**E**) Human umbilical vein endothelial cells (HUVECs) cultured in the supernatant of YTHDF1-silenced LoVo and SW480 cells exhibited significantly reduced angiogenic capacity, highlighting the critical role of YTHDF1 in angiogenesis. For Fig. 4A, B, one-way ANOVA was performed and for Fig. 4E, Student’s t-test was used to calculated significance. Statistical significance is indicated as * *P* < 0.05; ** *P* < 0.01, *** *P* < 0.001 to represent significant differences between different treatment groups

Initially, we verified YTHDF1 expression in various CRC cell lines and then selected the Lovo and SW480 cell lines for in vitro experiments (Fig. 4A). To effectively silence YTHDF1, we synthesized si-RNA targeting YTHDF1 and validated the effectiveness of si-YTHDF1#, si-YTHDF2#, and si-YTHDF3# using qPCR. Among these, si-YTHDF1# demonstrated the highest silencing efficiency (Fig. 4B), which led us to use si-YTHDF1 for further experiments.

Subsequently, we investigated the cellular localization of YTHDF1 using immunofluorescence, revealing its major role within the cell nucleus. Following YTHDF1 knockdown, a significant decrease in nuclear fluorescence and an increase in cytoplasmic fluorescence were observed (Fig. 4C). Additionally, to assess the impact of YTHDF1 silencing on angiogenesis, we performed VEGFa staining to label blood vessels. The results showed a significant reduction in angiogenesis in colorectal cancer upon YTHDF1 gene knockout, and this difference was statistically significant (Fig. 4D-E).

In summary, our study elucidated the crucial role of YTHDF1 in angiogenesis in colorectal cancer, particularly emphasizing its nuclear function in the angiogenesis process. These findings provide important biological evidence for future cancer therapeutic strategies targeting YTHDF1.

### The promoting effect of YTHDF1 on growth and angiogenesis of colorectal cancer in a xenograft mouse model

Investigating the Role of YTHDF1 in Colorectal Cancer (CRC) Development, Particularly its Effects on Tumor Growth and Angiogenesis, this research constructed two groups of CRC xenograft tumor models. The establishment of these models aimed to validate the impact of YTHDF1 expression level changes on CRC angiogenesis and proliferation.

**Fig. 5:**
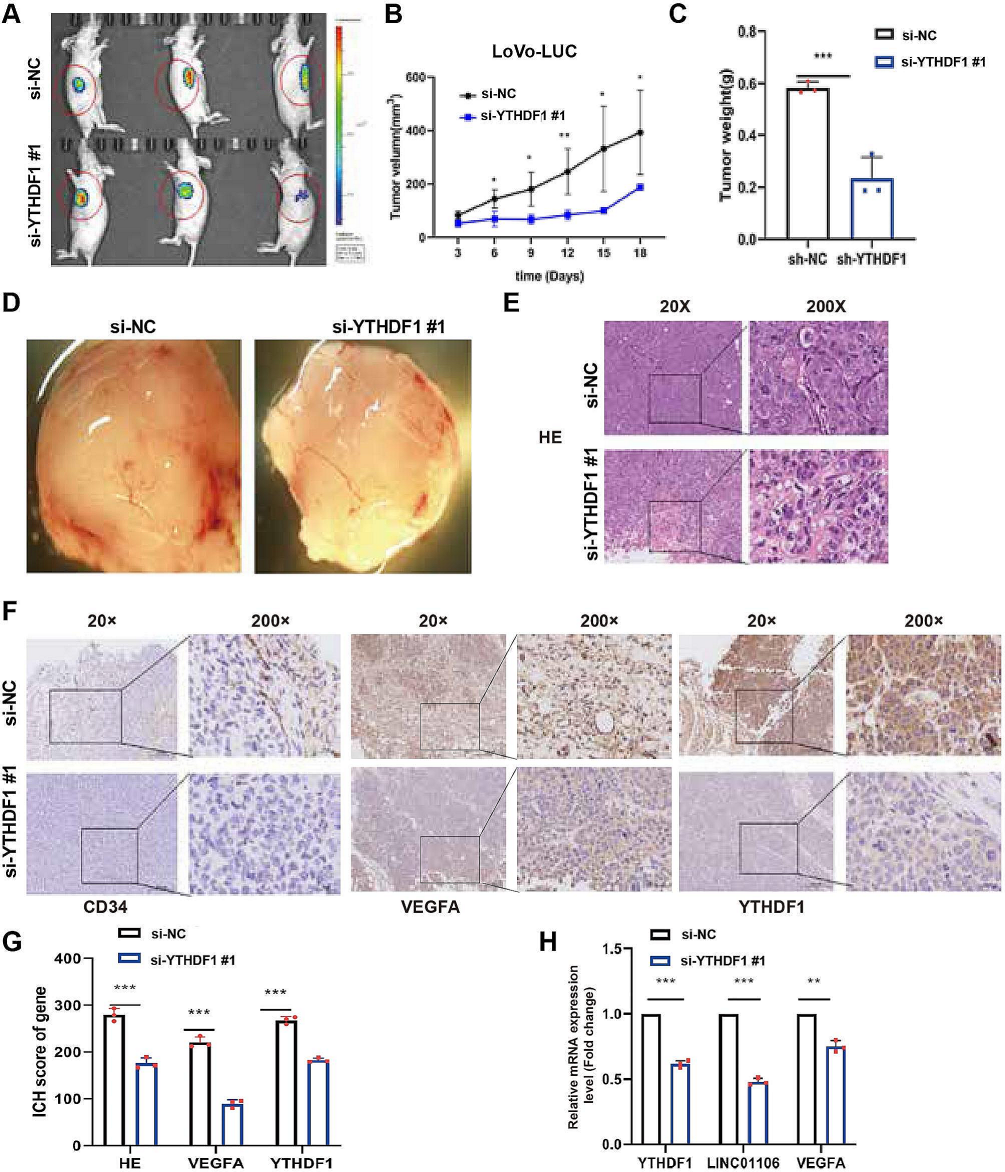
YTHDF1 promotes angiogenesis in CRC in vivo. Note: (**A**) Representative fluorescence intensity image in the vascular tumor model after subcutaneous injection of LoVo cells. (**B**) Changes in subcutaneous tumor growth volume following YTHDF1 knockdown. (**C**) Changes in tumor mass after YTHDF1 knockdown. (**D**) Representative images of angiogenesis observed under a stereomicroscope. (**E**) Subcutaneous tumors were stained with hematoxylin and eosin (**H**&**E**) to detect histological changes (scale bars: 200 μm and 20 μm). (**F**-**G**) Expression levels of VEGFA, CD34, and YTHDF1 in subcutaneous tumor tissues were examined using immunohistochemistry. (**H**) RNA was extracted from pulverized samples to detect the expression of YTHDF1, LINC01106, and VEGFA. For Fig. 5B, C, G, H, Student’s t-test was used to calculated significance and statistical significance is indicated as * *P* < 0.05; ** *P* < 0.01, *** *P* < 0.001 to represent significant differences between different treatment groups

Fluorescence measurements of the tumors in both groups of CRC xenograft models were conducted using a bioluminescence system (Fig. 5A). The research findings demonstrated that downregulation of YTHDF1 expression significantly reduced tumor volume and mass (Fig. 5B-C). Additionally, the downregulation of YTHDF1 expression also inhibited tumor angiogenesis (Fig. 5D). Immunohistochemistry (IHC) staining confirmed the decreased expression of YTHDF1 in the si-YTHDF1-1 group (Fig. 5E). IHC results further validated the downregulation of VEGFa, YTHDF1, and CD31 expression levels in the si-YTHDF1-1 group (Fig. 5F-G). The downregulation of VEGFa, YTHDF1, and LINC01106 expression levels in the si-YTHDF1-1 group was verified through Q-PCR technology (Fig. 5H).

In summary, the results of this study clearly indicate that YTHDF1 plays a critical promoting role in the growth and angiogenesis of colorectal cancer. The downregulation of YTHDF1 expression not only significantly inhibits tumor volume and mass but also reduces tumor angiogenesis, revealing the importance of YTHDF1 as a potential therapeutic target for colorectal cancer treatment.

### Functional validation of the YTHDF1/LINC01106/VEGFA axis in colorectal cancer angiogenesis

To delve into the mechanism by which YTHDF1 regulates the expression of LINC01106 through an m6A-dependent pathway and further validate the role of the YTHDF1/LINC01106/VEGFA axis in colorectal cancer (CRC) angiogenesis, a series of rescue experiments were designed in this study. These experiments included YTHDF1 knockout, LINC01106 overexpression, and the use of angiogenesis inhibitors.

**Fig. 6:**
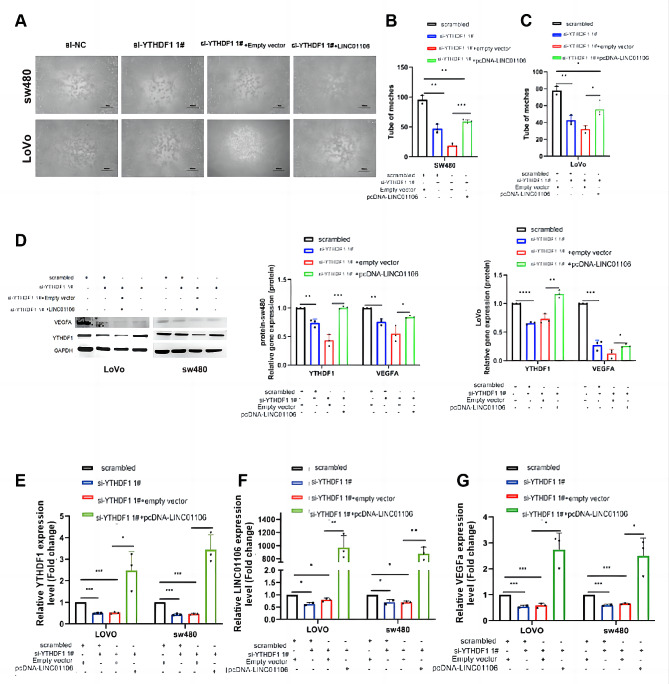
Restoration of angiogenic capacity by LINC01106 under YTHDF1 knockout background. Note: (**A**) YTHDF1 knockout experiments were performed in LoVo and sw480 cells, followed by tube formation assays to assess angiogenic capacity and observe the effect of LINC01106 overexpression. (**B**-**C**) Quantitative analysis of the formed tube numbers was conducted using image analysis software to evaluate the impact of LINC01106 overexpression on angiogenesis. (**D**) The protein expression levels of YTHDF1 and VEGFA were analyzed by Western blotting to investigate the effects of YTHDF1 knockout and LINC01106 overexpression. (**E**-**G**) Real-time quantitative PCR (RT-PCR) was employed to detect the influence of YTHDF1 knockout and LINC01106 overexpression on the mRNA expression levels of YTHDF1, LINC01106, and VEGFA. For Fig. 6B, C, D, E, F, G, one-way ANOVA was performed and statistical significance is indicated as * *P* < 0.05; ** *P* < 0.01, *** *P* < 0.001 to represent significant differences between different treatment groups

Firstly, the impact of YTHDF1 knockout on the angiogenic capability of CRC cells was evaluated. Subsequently, the effects of introducing the LINC01106 overexpression plasmid on the angiogenic capability of SW480 and Lovo cells treated with si-YTHDF1 were assessed. The experimental results demonstrated that LINC01106 overexpression significantly enhanced angiogenesis (Fig. 6A-C). Moreover, the consistency of these findings was confirmed by Western blot analysis (Fig. 6D), and RT-PCR results further validated the synergistic role of YTHDF1, LINC01106, and VEGFA in CRC angiogenesis (Fig. 6E-G).

In summary, the results of this study clearly reveal that YTHDF1 regulates LINC01106 in an m6A-dependent manner, thereby affecting the expression of VEGFA and playing a crucial role in colorectal cancer angiogenesis.

### LINC01106 targets miR-449b-5p to exert its angiogenic function in colorectal cancer

**Fig. 7:**
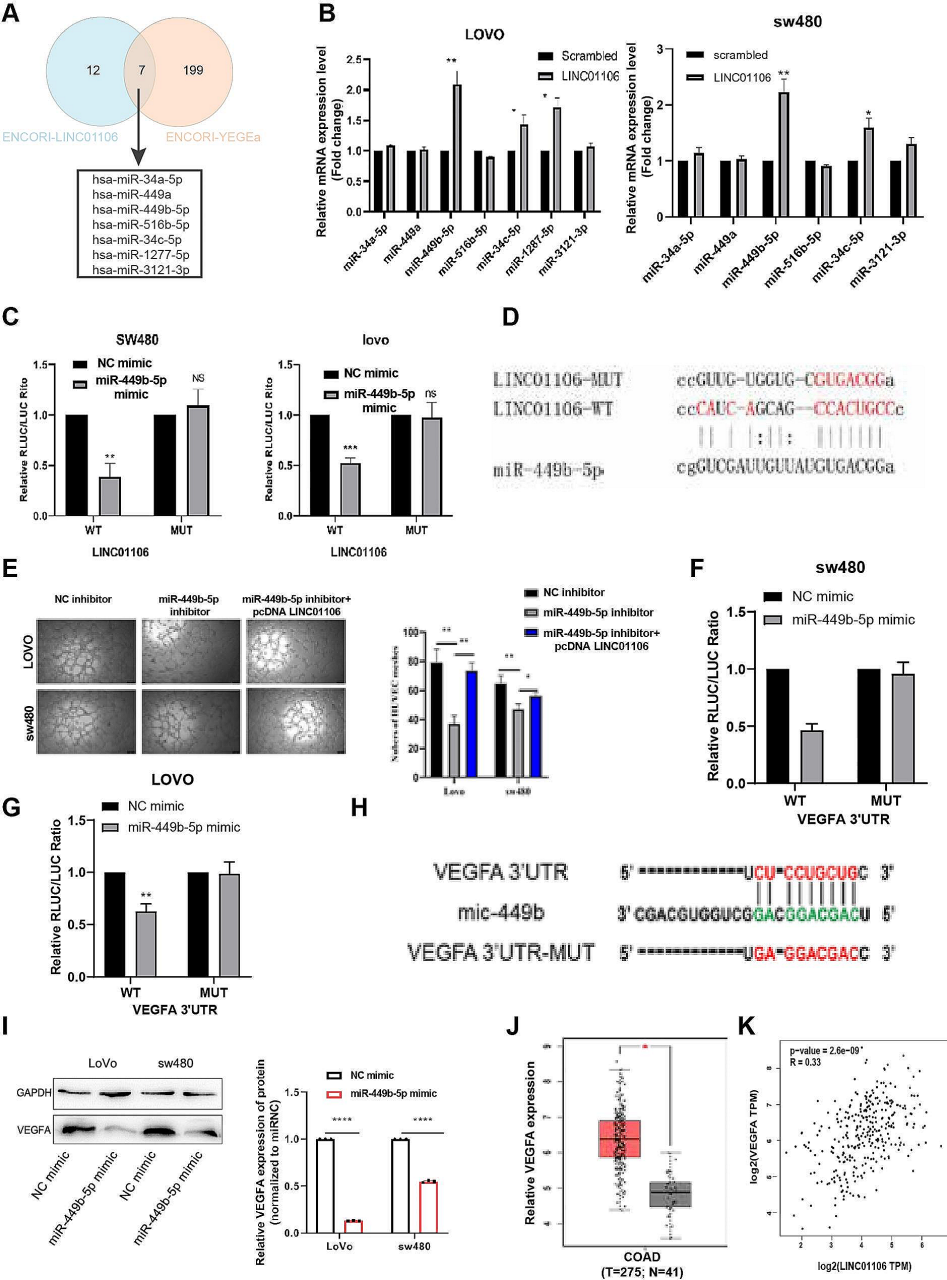
LINC01106 promotes angiogenesis in colorectal cancer by targeting miR-449b-5p. Note: (**A**) Prediction from the ECORI database revealed the presence of complementary sequences between LINC01106, VEGFA, and seven miRNAs. (**B**) AGO2-RIP experiments showed the enrichment of these seven predicted miRNAs in SW480 and LoVo cells overexpressing LINC01106 or treated with Scramble. (**C**) In the dual luciferase reporter assay, cells SW480 and LoVo were co-transfected with fluorescent reporter gene vectors containing LINC01106 or its mutant transcript and miR-449b-5p, followed by measuring luciferase activity. The results are presented as the relative ratio of Renilla luciferase activity to Firefly luciferase activity. (**D**) The potential binding sites between LINC01106 and miR-449b-5p (**E**) Tube Formation Assay results exhibited the proliferation of SW480 and LoVo cells co-transfected with miR-449b-5p mimics and pcDNA-LINC01106. Magnification: 100x, scale bar: 100 μm. (**F**-**G**) The luciferase activity assay in SW480 and Lovo cells co-transfected with fluorescent reporter gene vectors containing VEGFA or its mutant transcript and miR-449b-5p. The results are presented as the relative ratio of Renilla luciferase activity to Firefly luciferase activity. (**H**) Potential binding sites between VEGFA and miR-449b-5p. (**I**) After treating SW480 and Lovo cells with the miR-449b-5p mimic, the levels of VEGFA mRNA and protein were examined. (**J**) Analysis of the TCGA database reveals upregulation of VEGFA expression in colorectal cancer samples. (**K**) The expression of LINC01106 is positively correlated with VEGFA expression (*P* = 2e-9). Data represent the mean ± SD from three independent experiments. For Fig. 7B, C, F, G, I, Student’s t-test was used to calculated significance and for Fig. 7E, one-way ANOVA was performed. Statistical significance is indicated as * *P* < 0.05; ** *P* < 0.01, *** *P* < 0.001 to represent significant differences between different treatment groups

To investigate the mechanisms of LINC01106 in colorectal cancer (CRC), this study initially identified miRNAs with potential complementary binding sequences to LINC01106 using the ENCORI database. A total of 7 miRNAs with potential binding sequences were identified (Fig. 7A). It was observed that the overexpression of LINC01106 significantly increased the expression of miR-449b-5p in CRC cells (Fig. 7B). Luciferase reporter gene experiments indicated that the miR-449b-5p mimic markedly reduced luciferase activity in CRC cells with the wild-type LINC01106 sequence, whereas this effect was not observed in cells with the mutant LINC01106 sequence (Fig. 7C-D). Additionally, when miR-449b-5p inhibitor was introduced into SW480 and LOVO cells, the angiogenic capacity of tumor cells decreased. However, this capability was significantly enhanced when LINC01106 was overexpressed again (Fig. 7E).

Further luciferase reporter gene experiments demonstrated that the miR-449b-5p mimic significantly reduced the luciferase activity of wild-type VEGFA sequence in CRC cells, while no significant difference was observed in cells with mutant VEGFA sequences (Fig. 7F-G-H). Western blot results indicated that the overexpression of miR-449b-5p led to a decrease in VEGFA expression levels (Fig. 7I). Finally, analysis of TCGA database revealed a significant elevation in VEGFA expression levels in colorectal cancer patients (Fig. 7J), showing a positive correlation between LINC01106 and VEGFA in colorectal cancer (Fig. 7K).

These findings suggest that LINC01106 promotes CRC angiogenesis through targeting miR-449b-5p, providing new potential targets for colorectal cancer therapeutics.

## Discussion

There is increasing evidence to suggest that lncRNAs play a crucial role in the progression of cancer. Specifically, lncRNAs have been shown to regulate the proliferation and differentiation of endothelial cells [55]. For instance, lncRNA MALAT1 promotes endothelial cell proliferation and angiogenesis, whereas lncRNA H19 inhibits endothelial cell proliferation and vascular formation [56, 57]. Additionally, lncRNAs are believed to be involved in signaling pathways associated with angiogenesis. For example, lncRNA ANRIL promotes angiogenesis by modulating the VEGF signaling pathway (18). Moreover, lncRNA HOTAIR regulates the breakdown of extracellular matrix during angiogenesis, thereby facilitating endothelial cell migration and vascular formation [42, 58]. In this study, we employed TCGA data to identify differentially expressed lncRNAs in CRC. We found that LINC01106 was significantly upregulated in CRC and was associated with poorer patient prognosis. Furthermore, we confirmed the high expression of LINC01106 in CRC using RT-qPCR.

A higher level of LINC01106 is associated with various malignant clinical pathological features and serves as an independent predictive factor for adverse survival outcomes. Through loss-of-function and gain-of-function experiments, we demonstrated that LINC01106 promotes proliferation, migration, and angiogenesis in CRC cells in vitro. Our study reveals that LINC01106 exhibits significant pro-angiogenic functions. In HUVEC cells treated with CRC cell suspensions, depletion of LINC01106 suppressed tumor angiogenesis and growth. The xenograft mixed gel model has become a valuable preclinical model for tumor neovascularization. We utilized the xenograft mixed gel model to validate the tumorigenic effect of LINC01106 in vivo.

The isolation of miRNAs is the most commonly reported mechanism for lncRNAs to exert their regulatory function [59]. Considering the cytoplasmic distribution of LINC01106 in CRC, studies have reported that LINC01106 can act as a miRNA sponge [60]. We screened six overlapping miRNAs in two different bioinformatics databases and validated the binding between LINC01106 and miR-449b-5p using dual-luciferase reporter gene and RIP analysis. Further functional experiments indicated that LINC01106 sequestering miR-449b-5p promotes the progression of colorectal cancer. Previous studies have shown that igf2bp2-induced circRUNX1 promotes the growth and metastasis of esophageal squamous cell carcinoma through the miR-449b-5p/FOXP 3 axis [61]. miR-449b-5p has also been demonstrated to have tumor-suppressive roles in various types of malignant tumors, including pancreatic cancer [62], colorectal cancer [63], cervical cancer [64], glioblastoma [65], and lung cancer [66]. To investigate the target genes of the LINC01106/miR-449b-5p pathway in CRC, we combined four bioinformatics algorithms and RNA sequencing results and identified VEGFA as the downstream effector gene of the LINC01106/miR-449b-5p axis. VEGF has been recognized as a gene that promotes angiogenesis in many cancers [67, 68]. Currently, anti-vascular therapy drugs for advanced colorectal cancer, such as bevacizumab, have been developed [69]. Our research findings demonstrate that LINC01106 promotes tumor angiogenesis through the miR-449b-5p/VEGFA pathway. LINC01106 enhances the level of VEGFA and facilitates the formation of tumor blood vessels. Our data provide new insights into the role of LINC01106 in neovascularization in CRC.

To the best of our knowledge, this is the first study on the co-regulation of lncRNA and mRNA by m6A in CRC angiogenesis. Firstly, accumulating data suggests that YTHDF1, serving as an m6A “reader,” is upregulated in almost all cancers [45, 70]. The upregulation of YTHDF1 indicates a poorer prognosis for CRC patients [71]. It has been reported that YTHDF1 is upregulated in hepatocellular carcinoma due to regulation by HIF1α [72]. However, its clinical significance in colorectal cancer is still unclear. Here, we discovered that, in line with previous research, higher levels of YTHDF1 are associated with worse survival outcomes in CRC, and elevated expression of YTHDF1 is a prognostic factor. According to previous studies, the loss of YTHDF1 promotes tumor angiogenesis and influences its function in CRC treatment with HUVEC. These findings may provide an explanation for the upregulation of YTHDF1 in cancer but further investigation is needed specifically in CRC.

In terms of function, YTHDF1 demonstrates a dual role in cancer. It can inhibit the growth, invasion, and metastasis of various malignant tumors, but it can also accelerate tumor progression [73, 74]. Previous research has indicated that YTHDF1 can suppress proliferation and invasion by inhibiting [75]. In this study, we further discovered that overexpression of YTHDF1 promotes both in vitro and in vivo growth and angiogenesis in CRC cells, while depletion of YTHDF1 has the opposite effect. These findings suggest that YTHDF1 may act as an oncogenic driver in colorectal cancer.

In terms of mechanism, YTHDF1 is involved in tumor angiogenesis not only through the modification of m6A-dependent mRNA [72] but also through the modification of miR-375 or lncRNA XIST [76]. Previous studies have indicated that METTL14-mediated circGFRα1 facilitates self-renewal of female germ stem cells [77]. Here, we utilized m6A-lncRNA profiling and Me-RIP to identify the m6A-dependent modification of LINC01106 by YTHDF1 in CRC cells. Knockdown of YTHDF1 led to a decrease in m6A levels but also a reduction in the expression of LINC01106. YTHDF1 depletion suppressed vascular formation in CRC cells and weakened the stability of LINC01106. Our findings reveal the potential role of YTHDF1 in mediating the m6A-dependent modification of LINC01106.

The results of this study provide new insights into the molecular mechanisms underlying CRC. Specifically, the role of m6A modification of LINC01106 and the miR-449b-5p-VEGFA signaling pathway in regulating vascular formation in CRC offers a novel perspective on studying tumor microenvironment and angiogenesis. Furthermore, these findings contribute to the understanding of the widespread impact of m6A modification in cancer, especially in regulating lncRNA function. Therefore, this study not only establishes a new theoretical foundation for CRC research but also suggests potential research directions for other types of cancer.

From a clinical perspective, the findings of this study provide novel targets for the diagnosis and treatment of CRC. The expression levels of LINC01106 and YTHDF1 may serve as potential biomarkers for the prognosis of CRC patients. Additionally, interventions targeting the m6A modification of LINC01106 or the miR-449b-5p-VEGFA signaling pathway could provide a basis for the development of new therapeutic strategies. In the future, these findings have the potential to be translated into new diagnostic tools or treatment approaches, thereby improving the therapeutic efficacy and survival rate of CRC patients.

Despite providing crucial insights into the role of LINC01106 and YTHDF1 in CRC, this study has certain limitations. Firstly, the sample size of this study was relatively small, and it is important to validate these findings in larger sample sets. Secondly, this study primarily focused on cell and animal models, and future research should include more clinical samples and data. Additionally, while this study primarily focused on LINC01106 and YTHDF1, future investigations could explore other molecules that interact with these factors to gain a deeper understanding of the complex molecular networks in CRC. Furthermore, utilizing the weighted gene co-expression network analysis (WGCNA) method could help identify gene co-expression networks in CRC tissues [78], facilitating the discovery of hub genes and further validating the co-expression relationship between LINC01106 and YTHDF1 in CRC.

## Conclusions

**Fig. 8:**
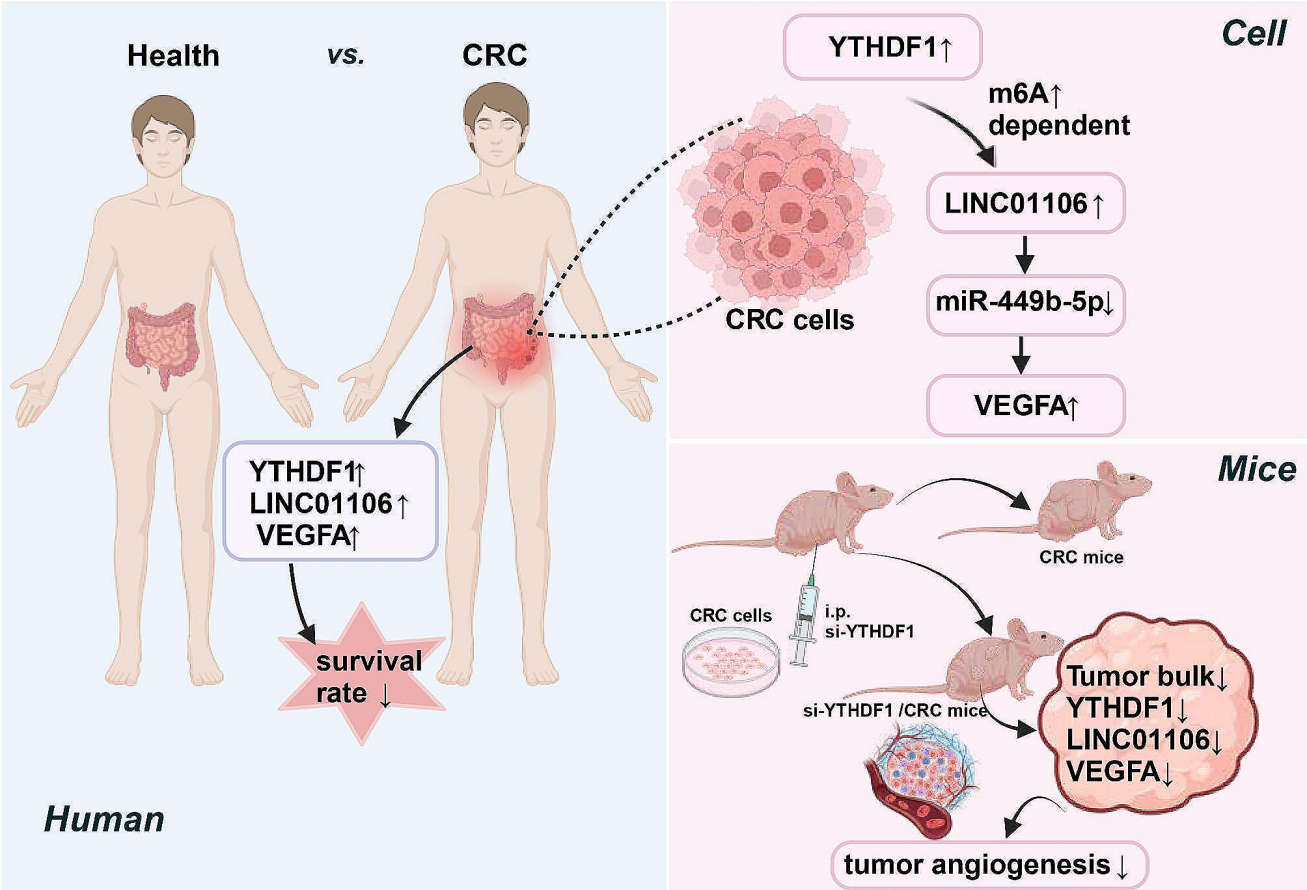
The role of LINC01106 in colorectal cancer, depicting its influence on angiogenesis through the m6A modification and the miR-449b-5p-VEGFA signaling pathway mechanism

In summary, this study elucidates the role of LINC01106 in colorectal cancer (CRC), particularly its impact on CRC angiogenesis through the m6A modification and the miR-449b-5p-VEGFA signaling pathway (Fig. 8). These findings not only enhance our understanding of the molecular mechanisms of CRC but also provide a theoretical foundation for the development of novel therapeutic strategies. From a clinical perspective, these discoveries hold promise for application in the diagnosis and treatment of CRC, thereby improving patient prognosis. Future research efforts should focus on validating the clinical relevance of these findings and exploring new therapeutic approaches based on these mechanisms. With these efforts, we can aspire to make significant progress in the treatment and management of CRC.

### Electronic supplementary material

Below is the link to the electronic supplementary material.

Supplementary Material 1

## Data Availability

No datasets were generated or analysed during the current study.

## Declarations

Animal Studies: All experiments that used animals were approved by the Institutional Animal Care and Use Committee of Nanjing Normal University (Nanjing, China). Authorization: IRB#20200047. (Approval number: IACUC-20200515)

## Abbreviations

CRC: colorectal cancer
LncRNAs: long non-coding RNAs
FBS: fetal bovine serum
ECM: endothelial cell medium
CDS: coding sequences
