# Supplementary figures and images for: YTHDF1’s grip on CRC vasculature: insights into LINC01106 and miR-449b-5p-VEGFA axis
Source: Cancer Cell Int. 2024 Jun 4;24:195. doi: 10.1186/s12935-024-03360-y (PMC11149289; doi:10.1186/s12935-024-03360-y)

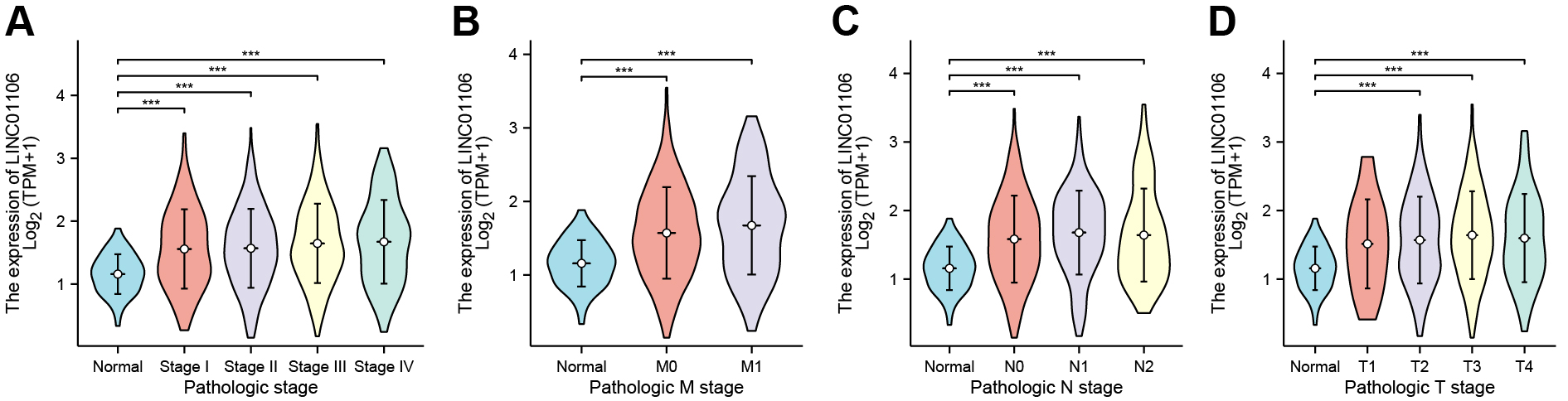

Supplement: Supplementary file 1 — Supplementary Material 1 [file 12935_2024_3360_MOESM1_ESM.jpg]
